# Nano‐plastics disrupt systemic metabolism by remodeling the bile acid–microbiota axis and driving hepatic–intestinal dysfunction

**DOI:** 10.1002/imt2.70103

**Published:** 2025-12-31

**Authors:** Yi Zhang, Zheng Lin, Runtong Huang, Yang Zhang, Lei Wang, Zan Fu, Chao Wang, Shuai Xiao, Heru Pramono, Kui Xu, Zhimin Xu, Yulong Yin

**Affiliations:** ^1^ Changsha Research Station for Agricultural & Environmental Monitoring, National Engineering Laboratory for Pollution Control and Waste Utilization in Livestock and Poultry Production, Institute of Subtropical Agriculture Chinese Academy of Sciences Changsha 410125 China; ^2^ Department of Colorectal Surgery The First Affiliated Hospital of Nanjing Medical University Nanjing 210000 China; ^3^ Department of Anesthesiology The First Affiliated Hospital of Nanchang University Nanchang 330006 China; ^4^ College of Environmental Science and Engineering Nankai University Tianjin 300350 China; ^5^ Nanjing Medical University Nanjing 211166 China; ^6^ Universitas Airlangga Mulyorejo Street Surabaya East Java 601115 East Java Indonesia

## Abstract

The pervasiveness of microplastic pollution poses a growing health risk, yet its long‐term metabolic consequences remain poorly defined. Here, we exposed mice to polyethylene terephthalate nanoparticle (NP) and combined histopathology, biochemistry, metabolomics, and metagenomics to resolve their interactions. NP ingestion induced a severe systemic phenotype characterized by weight loss, organ atrophy, dyslipidemia, and gut barrier collapse. Mechanistically, NPs disrupted bile acid (BA) homeostasis by hyperactivating hepatic synthesis pathways while suppressing microbial 7α‐dehydroxylation. This accumulation of cytotoxic BAs drove hepatic lipogenesis and aggravated mucosal inflammation. Crucially, metagenomics uncovered significant gut microbiota dysbiosis, where the enrichment of bile salt hydrolase‐encoding taxa and depletion of 7α‐dehydroxylating clades reinforced this BA imbalance. Furthermore, the microbiota exhibited functional deterioration, shifting toward glycan degradation with a concurrent loss of antibiotic resistance genes, signaling reduced ecological resilience. These findings identify BA dysregulation and specific microbiota functional losses as primary drivers of NP‐induced systemic metabolic collapse.

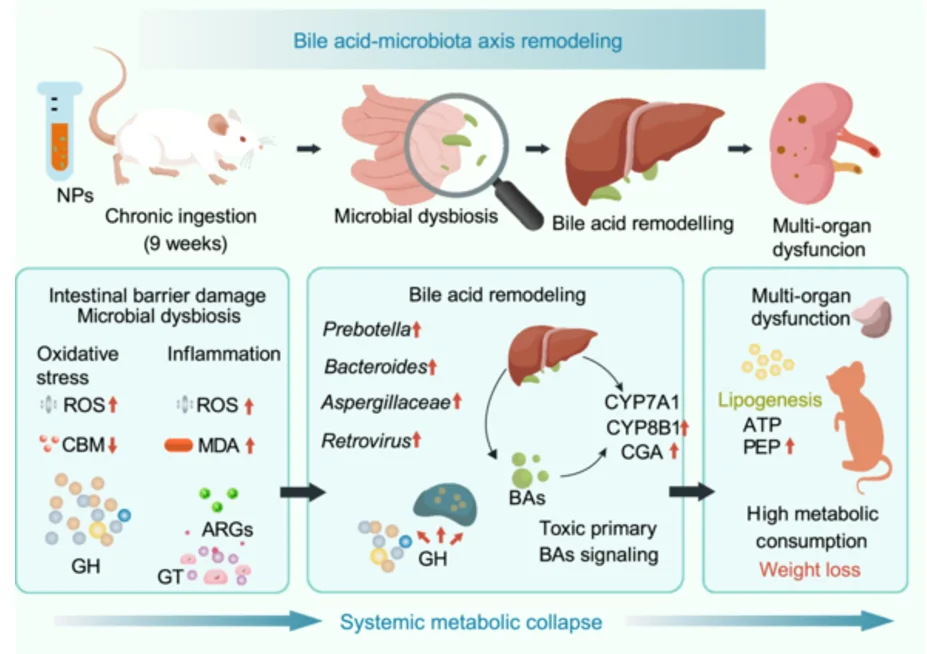

To the Editor,

Microplastic (MP) pollution has become globally pervasive, and nano‐sized particles (≤1 μm) are now detected in food, water, and indoor air, rendering human exposure nearly unavoidable. Their small size enables them to cross epithelial barriers, circulate systemically, and accumulate in distal organs such as the liver, kidney, and brain [1, 2]. Yet regulatory limits remain undefined because the mechanistic basis of their chronic organismal and molecular toxicity is still poorly understood.

MPs are known to induce oxidative stress, inflammation, apoptosis, genotoxicity, and disrupt mitochondrial, epigenetic, and endocrine pathways [3, 4]. However, most evidence centers on isolated organ injuries, leaving unclear how chronic exposure perturbs coordinated metabolic regulation. The gut–liver–kidney axis—critical for nutrient sensing and xenobiotic detoxification—is strongly shaped by gut microbiota, which govern lipid metabolism, bile acid (BA) transformation, redox balance, and immune homeostasis [2]. Microbial metabolites such as short‐chain fatty acids (SCFAs) and secondary BAs act through farnesoid X receptor (FXR) and G protein‐coupled receptor 5 (TGR5) to maintain metabolic stability, whereas dysbiosis drives metabolic disease [5]. Whether nanoplastics (NPs) dysregulate this inter‐organ microbe–metabolism network remains unknown. To address these issues, we established a 2‐month NPs exposure model and applied systems‐level analyses to construct an integrated “NPs–gut–microbiota–metabolism” framework, revealing how persistent exposure reprograms BAs signaling, microbial ecology, and systemic metabolic homeostasis.

## CHRONIC NPS EXPOSURE DISRUPTS GROWTH PERFORMANCE AND SYSTEMIC ENERGY METABOLISM

Institute of Cancer Research (ICR) mice were fed 200 nm‐NPs for long‐term exposure risk assessment, followed by histopathological, biochemical, metabolomic, and metagenomic analyses (Figure S1A). Behaviorally, exposed mice remained hyperactive during the day (Figure S1B). Because NPs may cross the gut–vascular barrier and accumulate in the hypothalamus, disrupting neurotransmitter turnover in orexinergic and catecholaminergic nuclei [2], this arousal likely reflects central involvement. Mechanistically, NPs may increase dopamine/norepinephrine synthesis, limit their degradation, and enhance glutamatergic signaling, thereby activating cAMP–PKA pathways and ion‐channel phosphorylation (HCN, Na⁺, Ca²⁺) to sustain neuronal firing [5]. Food intake declined while water intake increased (Figure S1C), and reduced nutrient availability combined with elevated expenditure led to weight loss after Week 5 (Figure S1D). Colon shortening and reduced mass (–21.28%, –30.30%; Figure S1E) suggest chronic inflammation and epithelial atrophy that impair absorption and enteroendocrine signaling [2], further suppressing appetite. Liver and kidney mass also decreased (–27.46%, –38.24%; Figure S1E), indicating catabolic remodeling. Serum biochemistry revealed systemic metabolic disruption: plasma glucose (+1.48‐fold), triglyceride (TG) (+4.17‐fold), total cholesterol (TC) (+1.79‐fold), pyruvate (PYR) (+1.23‐fold), and total bile acids (TBA) (+1.92‐fold) increased (Figure S1F), consistent with impaired hepatic lipid metabolism and perturbed FXR–SHP signaling [5]. Arginase (ARG) activity rose (+2.76‐fold), diverting arginine from nitric oxide synthase, reducing nitric oxide, worsening insulin sensitivity and microvascular control, and aggravating hyperglycemia and renal concentrating defects [6].

Targeted metabolomics showed broad re‐routing of energy flow: flavin adenine dinucleotide (FAD), guanosine monophosphate (GMP), guanosine triphosphate (GTP), and nicotinamide adenine dinucleotide phosphate (NADP) declined, while adenosine triphosphate (ATP), adenosine diphosphate (ADP), adenosine monophosphate (AMP), nicotinamide adenine dinucleotide (NADH), cyclic adenosine monophosphate (cAMP), phosphoenolpyruvate (PEP), and pyruvate increased (Figure S1G), indicating constrained mitochondrial oxidative phosphorylation—especially complex II—with compensatory glycolysis and gluconeogenesis [7]. Elevated ATP/ADP/AMP indicates accelerated substrate‐level phosphorylation; increased PEP and GDP with reduced GTP suggests PCK1‐driven gluconeogenesis. NADP depletion marks heightened oxidative stress with NADPH diverted to antioxidant defense, while 2‐oxoacid accumulation indicates a pyruvate dehydrogenase (PDH) redox block [8]. Reduced GMP and elevated cyclic guanosine monophosphate (cGMP) suggest guanylate reallocation to second‐messenger signaling, amplifying natriuretic and G protein coupled receptor (GPCR) pathways that intersect water–salt balance and BAs signaling [5, 9].

## NPS‐INDUCED HEPATIC–RENAL LIPID REMODELING VIA INFLAMMATION–METABOLISM CROSSTALK

NPs provoked pronounced hepatic–renal remodeling. AB‐PAS staining showed hepatocyte shrinkage, reduced lipid vacuoles, cytoplasmic loosening, fatty degeneration with eosinophilic bodies, lymphocytic infiltration, and congestion relative to orderly controls (Figure 1A). Hepatic TC and TG increased (2.18‐fold, 2.08‐fold), paralleled by renal TC and TG accumulation (TC +41.35%; TG +100.9%) (Figure 1B–E). Hepatic and renal TC/TG correlated positively (Figure S2), and blood TC and TG also rose (Figure S1F), indicating impaired hepatic lipid handling as a central driver of systemic dyslipidemia. The coordinated repression of *Chrebp*, *Gk*, and *Pkm* suggests a glucose‐insensitive, low‐glycolytic hepatic state, while the induction of *Fas*, *Gpat*, *Dgat1*, and *Dgat2* drives de novo lipogenesis and triglyceride packaging [10]. Coupled with down‐regulated *Pparα* and *Cpt1α*, this profile reflects a shift toward lipid storage with compromised mitochondrial fatty‐acid oxidation (Figure 1F). Elevated serum TBAs and ARGs (Figure S1F) indicate hepatocellular stress, disrupted nitrogen–lipid coupling, and perturbed FXR–SHP and SREBP1c feedback, linking TBA imbalance to cholesterol overload [5].

**Figure 1:**
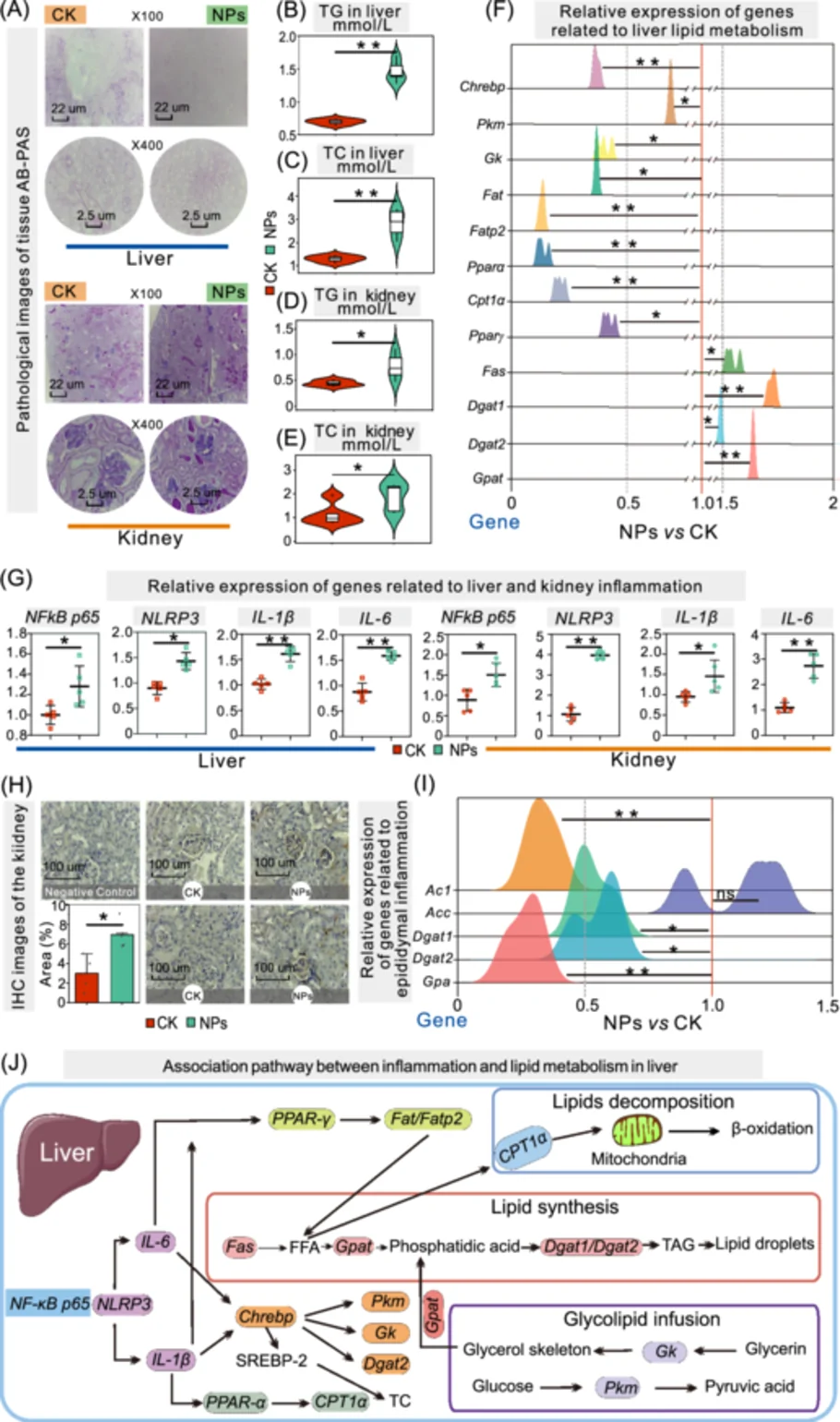
Chronic nanoparticles (NPs) exposure remodels hepatic and renal lipid metabolism and induces inflammatory responses. (A) Representative H&E‐stained liver and kidney sections after 9‐week NPs exposure (×100 and ×400 magnification). (B–E) Triglyceride (TG) and total cholesterol (TC) levels in liver and kidney tissues. (F) Relative hepatic expression levels of genes involved in glucose sensing, glycolysis, lipogenesis, triglyceride esterification, and fatty‐acid oxidation compared to the CK group (red line). Genes analyzed include *Chrebp*, *Pkm*, *Gk*, *Fat*, *Fatp2*, *PPAR‐α*, *CPT1α*, *PPAR‐γ*, *Fas*, *Dgat1*, *Dgat2*, and *Gpat*. (G) Relative expression of inflammatory markers (*NFkB p65*, *NLRP3*, *IL‐1β*, and *IL‐6*) in liver and kidney compared with the CK group. (H) Immunohistochemical staining of kidney tissues using anti‐CD68 antibody, with corresponding negative controls. (I) Relative expression of lipid metabolism–related genes in epididymal adipose tissue (*Ac1*, *Acc*, *Dgat1*, *Dgat2*, and *Gpa*) compared with the CK group (red line). (J) Schematic illustration summarizing NPs‐induced crosstalk between inflammatory signaling and hepatic lipid metabolic pathways. Data represent biological replicates and are shown as means (*n* = 5). *p < 0.05, **p < 0.001, compared with controls. Statistical significance was determined using two‐tailed Student's *t*‐test (two‐group comparisons).

Inflammation mirrored metabolic injury. *NF‐κB p65*, *NLRP3*, *IL‐1β*, and *IL‐6* rose 1.14–4.09‐fold in liver and kidney (Figure 1G), with renal *Caspase‐3* elevation (Figure 1H). Renal *NF‐κB*/*NLRP3*/*IL‐6* correlated with hepatic *Dgat1*/*Dgat2*/*Gpat* (Figure S2), highlighting inflammation‐driven inter‐organ coupling. Mechanistically, *NF‐κB* inhibits *Pparα*‐dependent β‐oxidation, while inflammasome *IL‐1β* suppresses *Pparα*/*Cpt1α* and activates SREBP1*c*, diverting acetyl‐CoA to DGAT‐mediated TG storage [3, 8]. BA dysregulation further enhances lipid accumulation via FXR–SHP and SREBP1c crosstalk [5]. In epididymal adipose tissue, *Acl*, *Dgat1/2*, and *Gpa* were downregulated (–44.43% to –66.56%) except *Acc* (Figure 1I), indicating systemic suppression of peripheral lipogenesis and lipid redistribution toward hepatic storage. Thus, NPs drive hepatic–renal lipid remodeling through inflammation–metabolism coupling, where *NF‐κB*/*NLRP3* activation limits FAO, enhances lipogenesis, and reinforces DGAT‐dependent TG esterification (Figure 1J).

## OXIDATIVE–INFLAMMATORY CROSSTALK DISRUPTS INTESTINAL BARRIER AND MUCOSAL HOMEOSTASIS

NPs also accumulated along the intestinal epithelium (Figure S3A,B). AB‐PAS staining revealed goblet‐cell depletion, discontinuous mucus, shortened villi, and epithelial disorganization with lymphocytic infiltration and vacuolar degeneration (Figure S3C). Oxidative balance deteriorated: reactive oxygen species (ROS) (+1.36‐fold), malondialdehyde (MDA) (+1.42‐fold), and NO (+1.35‐fold) increased, while superoxide dismutase (SOD), catalase (CAT), glutathione (GSH), myeloperoxidase (MPO), and GSH‐Px decreased and oxidized glutathione (GSSG) rose (Figure S3D), indicating impaired detoxification of O₂^−^/H₂O₂ and hydroperoxides and enhanced ONOO^−^‐mediated protein/lipid damage [8, 11].

*Muc1* and *Retnlb* rose, whereas *Muc2*, *Muc3*, *Klf4*, and *Meprin‐β* declined (Figure S3E). Barrier‐gene responses were segment‐specific: ileal *ZO‐1*, *CLDN2*, *OCLN*, and *SLC15A1* increased, while the colon displayed broad downregulation and *ALP1* loss (Figure S3F). This likely reflects *Nrf2*‐mediated ileal reinforcement versus colonic *NF‐κB*/*NLRP3*‐driven collapse under higher microbial and oxidative burden [3, 5]. Inflammation reinforced dysfunction: *NF‐κB p65*, *NLRP3*, *IL‐1β*, *IL‐6*, and *TNF‐α* were strongly increased in colon and moderately in ileum (Figure S3F). Correlation analysis (Figure S3G) supports a coherent mechanism: NPs suppress antioxidants, elevate ROS and NO, impair mucus secretion, disrupt junctions, and amplify *NF‐κB*/*NLRP3* remodeling, forming a self‐reinforcing cycle of oxidative imbalance, mucus loss, barrier collapse, and cytokine‐driven inflammation [5].

## BAS PATHWAY PERTURBATION MEDIATES HEPATIC–INTESTINAL METABOLIC CROSSTALK

NPs reshaped the BA pool, enriching primary conjugated BAs, including 7‐ketoLCA, taurodeoxycholic acid (TDCA), allocholic acid (ACA), tauro‐α‐muricholic acid (T‐α‐MCA), glycoursodeoxycholic acid (GUDCA), taurohyocholic acid (THCA), taurocholic acid (TCA), taurochenodeoxycholic acid (TCDCA), glycocholic acid (GCA), α‐muricholic acid (α‐MCA), ursodeoxycholic acid (β‐UDCA), 6,7‐diketolithocholic acid (6,7‐diketoLCA), and cholic acid (CA). Conversely, NPs depleted unconjugated and secondary BAs, such as tauro‐β‐muricholic acid (T‐β‐MCA), chenodeoxycholic acid (CDCA), β‐muricholic acid (β‐MCA), deoxycholic acid (DCA), lithocholic acid (LCA), and LCA‐3S (Figure 2). Potential drivers include (i) adsorption or sequestration of cholesterol and BA intermediates, (ii) interference with hepatic *CYP7A1/CYP27A1* caused by oxidative and mitochondrial stress, and (iii) disruption of microbial deconjugation and 7α‐dehydroxylation [12]. Hepatically, elevated CA/TCA/GCA indicate hyperactivation of the classical *CYP7A1–CYP8B1* pathway, diverting cholesterol toward CA/TCA rather than CDCA‐derived species (Figure 2A). Reduced CDCA/β‐MCA suggests suppression of the alternative *CYP27A1–CYP7B1* arm, consistent with mitochondrial and oxidative inhibition of *CYP27A1* [5, 13]. The sharp reduction of DCA/LCA reflects loss of microbial 7α‐dehydroxylase activity. Thus, cholesterol is funneled toward CA/TCA while conversion to hydrophobic FXR/TGR5‐active secondary BAs is blocked [14]. Altered BAs flux activates SREBP1c and weakens FXR–SHP feedback, promoting hepatic lipogenesis. Reduced CDCA/LCA diminishes FXR/TGR5 signaling that normally suppresses *CYP7A1*, restrains inflammation, and supports β‐oxidation [13]. This aligns with the observed transcriptional reprogramming: *Fas* and *Gpat* induction drives fatty acid synthesis; and *Pparα*/*Cpt1α* downregulation suppresses β‐oxidation (Figure 1F). *DGAT1* upregulation further channels acetyl‐CoA into triglyceride esterification [6], culminating in hepatic TG accumulation.

**Figure 2:**
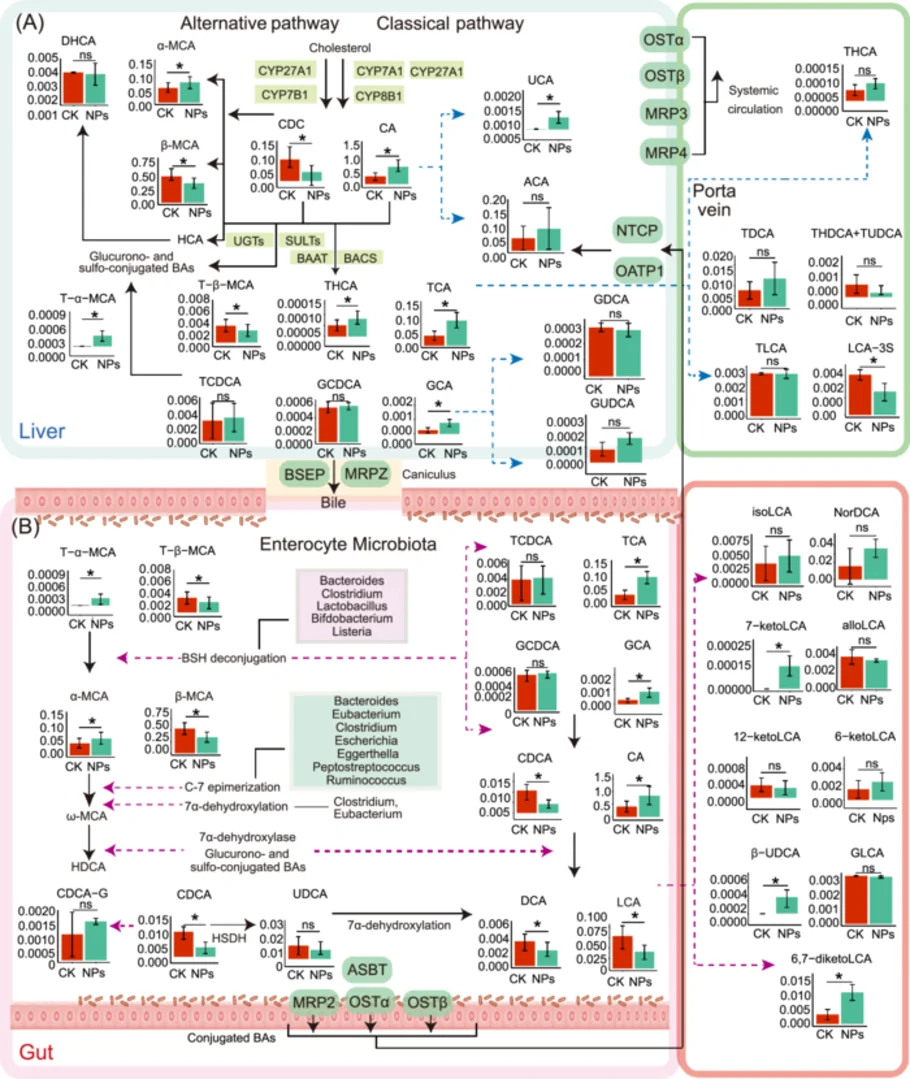
Chronic NPs exposure alters circulating bile acid composition and perturbs hepatic–intestinal metabolic pathways. (A and B) Bile acid profiles after 9‐week NPs exposure, showing relative changes in primary and secondary bile acids in liver (A) and gut (B). The pathway schematic illustrates hepatic bile acid synthesis via the classical *CYP7A1–CYP8B1* and alternative CYP27A1 pathways, as well as intestinal microbial biotransformation through 7α‐dehydroxylation. Solid lines and dashed lines, respectively represent the direct and indirect influence paths. Data represent biological replicates and are expressed as means (*n* = 5) in μg/mL. *p < 0.05, ^ns^ *p* > 0.05, compared with controls. Statistical significance was determined using two‐tailed Student's *t*‐test (two‐group comparisons).

Intestinal consequences are similarly profound. Loss of CDCA, DCA, and LCA—secondary BAs with epithelial‐protective and anti‐inflammatory activities—coincides with goblet cell depletion, reduced *Muc2* expression, and weakened mucus continuity. Enrichment of cytotoxic hydrophobic BAs (CA, 7‐ketoLCA, TDCA) intensifies epithelial injury and oxidative stress, elevating ROS/MDA and reducing SOD, CAT, GSH‐Px, and MPO while increasing NO (Figure 2B and S3D). Redox injury destabilizes tight junctions, producing compensatory upregulation in the ileum but collapse in the colon. BAs‐driven oxidative stress activates *NF‐κB p65* and *NLRP3* in the colon, increasing *IL‐1β*, *IL‐6*, and *TNF‐α* (Figure 1G). These cytokines further suppress hepatic *Pparα*/*Cpt1α* and reinforce DGAT‐mediated TG storage, integrating BA dysregulation with inflammation and lipid overload [15, 16]. Reduced LCA/CDCA also weakens FXR/TGR5‐dependent anti‐inflammatory signaling, locking the system into a pro‐inflammatory, lipogenic state [14, 17]. Although our multi‐dimensional data strongly support a major role of BAs remodeling in mediating hepatic–intestinal metabolic injury, causality remains to be validated. Future studies using BA sequestration, FXR/TGR5 modulation, or microbiota‐directed interventions are crucial to disentangle BA imbalance from direct toxicity.

## MICROBIAL DYSBIOSIS LINKS BAS REMODELING TO HEPATIC–INTESTINAL DYSFUNCTION

Metagenomic profiling revealed that NP exposure fundamentally restructured the gut ecosystem across bacterial, fungal, and viral domains. Bacterial communities exhibited a marked decline in α‐diversity (Figure S4) and a shift toward deterministic assembly (Figure S5A,B). Specifically, taxa abundance was significantly altered: carbohydrate‐fermenting genera such as *Prevotella* and *Bacteroides* were enriched, whereas SCFA‐producing and 7α‐dehydroxylation‐associated lineages like *Ruminococcus_sp.1xD2123* and *Lachnoclostridium_sp. An138* were depleted (Figures S6A, 7). LEfSe analyses identified shifts in *Prevotella, Bacteroides, and Alistipes* (Figure S8A and Table S1). This bacterial contraction strongly correlated with elevated oxidative stress markers (e.g., SOD, GSH, MDA, ROS) (Figure S9), reflecting a redox imbalance tied to the loss of beneficial microbes. Conversely, the fungal and viral biospheres expanded. Fungal populations showed increased diversity (Figure S10) and dispersal (Figure S5C,D), with enriched families like Aspergillaceae and Glomeraceae linking to sterol metabolism and ROS production (Figures S6B, 11). LEfSe analyses revealed significant abundance shifts in genera including *Puccinia*, *Lobosporangium*, *Erysiphe*, and *Trichoderma* (Figure S8B and Table S1). These fungal shifts negatively correlated with mucosal barrier integrity markers (e.g., *Muc1*/*2*/*3*, *Klf4*, *Meprin‐b*, *Retnlb*) (Figure S9), suggesting that fungal overgrowth exacerbates goblet cell dysfunction. Similarly, viral diversity increased with higher stochasticity (Figure S12). *Gammaretrovirus*, *Betaretrovirus*, and *T7virus* were enriched (Figures S6C, 8C). Phylogenetically identified taxa, including *Mouse mammary tumor virus* and *Microbacterium phage Lyell*, were mapped to families (e.g., Retroviridae, Siphoviridae) strongly correlated with *NF‐κB*/*NLRP3* signaling and epithelial junction markers (e.g., *SI*, *SLC15A1*) (Figures S9, 13). This indicates a viral mechanism driving *NF‐κB* activation, barrier disruption, and enhanced phage‐mediated gene transfer that exacerbates BA dysmetabolism [18].

Overall, enriched *Prevotella*/*Bacteroides* supply BSHs that deconjugate TCA/TCDCA, whereas loss of *Ruminococcus*/*Lachnoclostridium* reduces 7α‐dehydroxylation and impairs CA/CDCA conversion to DCA/LCA [16]. Fungal expansion (e.g., *Aspergillaceae*, *Glomeraceae*) elevates ROS and competes for cholesterol substrates, while retroviridae amplification disrupts tight junctions and BAs transporters (taurocholic acid co‐transport polypeptide, apical membrane sodium‐dependent bile salt transporter protein) [5, 14, 18]. NPs‐induced multi‐kingdom dysbiosis drives BAs remodeling, hepatic lipogenesis, intestinal barrier failure, and systemic inflammation.

## CAZY–ARG REMODELING CONNECTS MICROBIAL FUNCTIONAL LOSS WITH BAS–LIPID DYSREGULATION

Gene ontology (GO) analysis highlighted a strategic pivot toward defense responses and GPCR signaling (Figure S14 and Table S2), indicating intensified immunometabolic crosstalk where microbial metabolites (e.g., SCFAs, BAs) act as ligands to modulate enterohepatic circulation [5]. CAZy analysis further revealed a profound enzymatic restructuring from anabolic glycosylation to catabolic degradation: glycoside hydrolases (GHs) expanded to dominate the landscape (>40%) (Table S3), while glycosyltransferases (GTs) significantly declined (Figure S15A,B). Parallel increases in CEs (11.4% → 12.2%) and PLs (1.2% → 1.8%), contrasted with a decline in AAs (1.6% → 1.1%) (Figure S15C,D), reinforce a metabolic shift prioritizing aggressive polysaccharide cleavage over mucin‐related glycosylation. Specifically, the upregulation of GH1, GH81, and GH119—primarily mapped to *Muribaculaceae bacterium Isolate‐105* and *Firmicute*s *bacterium ASF500*—accelerates saccharide hydrolysis (Figure S15E). This functional shift increases monosaccharide availability, which fuels hepatic glycolytic‐lipogenic flux and drives triglyceride accumulation via *Chrebp* and DGAT1 [19]. Conversely, the suppression of GT8 and GT10 from mucin‐degrading specialists like *Akkermansia muciniphila* and *Prevotella sp*. impairs mucin O‐glycosylation, directly compromising epithelial defense [4]. Furthermore, despite a general CBM expansion, the specific loss of butyrate‐associated modules (CBM11, CBM71) deprives colonocytes of energy and weakens *NF‐κB* suppression, thereby amplifying intestinal inflammation (Figure S15F). Modest CE and PL increases (*Anaerotruncus*) further enhanced deacetylation and glycan cleavage, destabilizing microbial niches.

Concurrently, ARG profiling exhibited a distinct “contraction‐retention” pattern (Figure S15G–I). While broad‐spectrum resistance genes (*vanI*, *tetW*, *pp‐flo*) significantly decreased (Figure S15J and Table S4), the multidrug efflux pump *adeF* was upregulated (Figure S15K), indicating a survival strategy focused on retaining stress‐adaptive capacity. Mechanistically, this functional collapse establishes a pathogenic feed‐forward loop. The synergy of GH expansion, GT loss, and ARGs contraction creates a “high‐glucose, low‐SCFA” environment that erodes mucosal integrity and triggers ROS‐mediated inflammation [12, 20]. Overall, this drives a feed‐forward loop—mucus/SCFA loss → ROS/*NF‐κB* activation → hepatic–renal inflammation → deeper *PPARα*–*CPT1α* suppression and BAs dysregulation—linking microbial functional decline to triglyceride overload, BAs imbalance, renal oxidative stress, and intestinal barrier failure.

## AUTHOR CONTRIBUTIONS

**Yi Zhang**: Conceptualization; methodology; data curation; investigation; funding acquisition; project administration; writing—original draft; visualization; validation. **Zheng Lin**: Conceptualization; writing—review and editing. **Runtong Huang**: Software. **Yang Zhang**: Resources. **Lei Wang**: Data curation; investigation. **Zan Fu**: Formal analysis. **Chao Wang**: Writing—review and editing. **Shuai Xiao**: Data curation; investigation; validation. **Heru Pramono**: Writing—review and editing. **Kui Xu**: Data curation. **Zhimin Xu**: Methodology; conceptualization; validation; project administration; resources; supervision; writing—review and editing. **Yulong Yin**: Project administration; resources; supervision; writing—review and editing. All authors have read the final manuscript and approved it for publication.

## CONFLICT OF INTEREST STATEMENT

The authors declare no conflicts of interest.

## ETHICS STATEMENT

The ethics application (Approval No: IACUC‐2510049) for mouse study was approved by the CAM‐SU Animal Care and Use Committee of the Nanjing Medical University.

## Supporting information


**Figure S1.** Effects of chronic NPs exposure on growth performance, organ indices, and systemic metabolic profiles in mice.
**Figure S2.** Co‐occurrence network among TC and TG content, lipid metabolism‐related genes, and inflammatory factors in liver and kidney.
**Figure S3.** Chronic NPs exposure disrupts intestinal redox balance, barrier integrity, and inflammatory signaling.
**Figure S4.** Alpha diversity of intestinal bacterial communities in mice.
**Figure S5.** Neutral community models illustrating the contribution of stochastic processes to community assembly.
**Figure S6.** Heat‐tree visualizations showing differential taxonomic abundance of intestinal microbiota.
**Figure S7.** Chronic NPs exposure alters intestinal bacterial community structure in mice.
**Figure S8.** Linear discriminant analysis effect size identifying differentially enriched taxa.
**Figure S9.** Heatmap showing Spearman correlation coefficients between gut physicochemical indices and abundances of characteristic microbial taxa.
**Figure S10.** Alpha diversity of intestinal fungal communities in mice.
**Figure S11.** Chronic NPs exposure alters intestinal fungal community structure in mice.
**Figure S12.** Alpha diversity of intestinal viral communities in mice.
**Figure S13.** Chronic NPs exposure alters intestinal viral community structure in mice.
**Figure S14.** GO functional enrichment of intestinal microbiota in mice.
**Figure S15.** Effects of chronic NPs exposure on intestinal CAZymes and ARGs.
**Figure S16.** SEM and fluorescence images characterizing the tested NPs.
**Figure S17.** The dissection process and sample collection of mice after 9 weeks of exposure.

**Table S1.** Differentially enriched bacterial taxa identified by LEfSe analysis.
**Table S2.** Top 10 significantly up‐regulated and down‐regulated GO functional annotations.
**Table S3.** Taxonomic attribution of key differentially abundant genes from the CAZy database.
**Table S4.** Taxonomic attribution of key differentially abundant ARGs.
**Table S5.** List of plasma biochemical assay kits used in this study.
**Table S6.** List of enzymatic assay kits used in this study.
**Table S7.** Sequences of qPCR primers used in this study.

Ethical Approval.

## Data Availability

The data that supports the findings of this study are available in the supplementary material of this article. All the sequencing data have been deposited in NCBI under BioProject accession number PRJNA1362669 (https://www.ncbi.nlm.nih.gov/bioproject/PRJNA1362669). The data and scripts used are saved in GitHub (https://github.com/linzhengpro/Mouse). Supplementary materials (methods, figures, tables, graphical abstract, slides, videos, Chinese translated version and update materials) may be found in the online DOI or iMeta Science http://www.imeta.science/.
