# Supplementary material for: Nano‐plastics disrupt systemic metabolism by remodeling the bile acid–microbiota axis and driving hepatic–intestinal dysfunction
Source: Imeta. 2025 Dec 31;5(1):e70103. doi: 10.1002/imt2.70103 (PMC13726609; doi:10.1002/imt2.70103)
Supplement: Supplementary file 1 [file IMT2-5-e70103-s002.pdf]

动物实验伦理审查同意书
Affidavit of Approval of IACUC

申请编号<br>Application No.	23241	批准编号<br>Approval No.	IACUC-2510049

本《动物实验方案》经过实验动物管理与伦理委员会审核，符合动物保护、动物福利和伦理原则，符合国家动物福利伦理的相关规定。方案的相关信息如下

The animal use protocol listed below has been reviewed and approved by Institutional Animal Care and Use Committee (IACUC)

实验名称<br>Protocol Title	小鼠暴露微塑料后研究其对肝-肠轴胆汁酸代谢和脂质代谢以及宏基因组分析了肠道菌群的功能<br>Effects of microplastics exposure on liver and intestinal function and microbiota in mice				
申请人姓名<br>Applicant	谭思悦<br>Tan Siyue	职称/学位<br>Title/Degree	研究生<br>postgraduate	邮箱<br>Email	1781754229@qq.com
实验负责人<br>Principle Investigator	王超<br>Wang Chao	职称/学位<br>Title/Degree	博士/副教授<br>associate professor	邮箱<br>Email	wangchao@njmu.edu.cn
院系（部门）<br>Department	南京医科大学公共卫生学院<br>Nanjing Medical University, School of Public Health			申请日期<br>Application Date	2025-09-09
拟实验时间<br>Period of Protocol	2025-09-20<br>-<br>2027-09-23	实验动物使用许可证<br>Number of Animal Use Permit		SYXK(苏)2025-0025	
审核意见<br>Results of Inspection	符合动物福利伦理要求，可以进行实验。Agree				
兽医师<br>Chief Veterinary Officer	马贯中<br>Ma Guanzhong	马贯中 Ma Guanzhong		日期<br>Date	2025.12.14

南京医科大学实验动物福利伦理委员会
Institutional Animal Care and Use Committee of NMU

主席(chairman)：施爱民　签名(signature)：施爱民 Shi Aimin

Shi Aimin

IACUC OF NMU

日期(Date)：2025-9-10
