# Supplementary material for: Nano‐plastics disrupt systemic metabolism by remodeling the bile acid–microbiota axis and driving hepatic–intestinal dysfunction
Source: Imeta. 2025 Dec 31;5(1):e70103. doi: 10.1002/imt2.70103 (PMC13726609; doi:10.1002/imt2.70103)
Supplement: Supplementary file 2 [file IMT2-5-e70103-s001.docx]

**Supporting information to**

**Nano-plastics disrupt systemic metabolism by remodeling the bile acid–microbiota axis and driving** **hepatic–intestinal dysfunction**

**Running title:** Nanoplastics disrupt metabolism via bile acid–microbiota axis

Yi Zhang^1,2^, Zheng Lin^1^, Runtong Huang^1^, Yang Zhang^3^, Lei Wang^4^, Zan Fu^2^, Chao Wang^5^, Shuai Xiao^1^, Heru Pramono^6^, Kui Xu^1^, Zhimin Xu^1*^, Yulong Yin^1*^

^1^Changsha Research Station for Agricultural & Environmental Monitoring, National Engineering Laboratory for Pollution Control and Waste Utilization in Livestock and Poultry Production, Institute of Subtropical Agriculture, Chinese Academy of Sciences, Changsha 410125, China

^2^Department of Colorectal Surgery, The First Affiliated Hospital of Nanjing Medical University, Nanjing 210000, China

^3^Department of Anesthesiology, The First Affiliated Hospital, Nanchang University, Nanchang 330006, China

^4^College of Environmental Science and Engineering, Nankai University, Tianjin 300350, China

^5^Key lab of Modern Toxicology of Ministry of Education, Center for Global Health, School of Public Health, Nanjing Medical University, Nanjing 211166, China

^6^Laboratory of Fisheries Microbiology, Department of Marine Science, Faculty of Fisheries and Marine, Universitas Airlangga, Surabaya 601115, Indonesia

*Correspondence: [zhiminxusoil@isa.ac.cn](mailto:zhiminxusoil@isa.ac.cn) (Zhimin Xu); [yinyulong@isa.ac.cn](mailto:yinyulong@isa.ac.cn) (Yulong Yin)

**Supplementary method**

**Animals and housing conditions**

Male ICR mice (4 weeks old; initial body weight 26–28 g) were obtained from the Guangdong Provincial Laboratory Animal Center (Guangzhou, China). Animals were housed under specific pathogen-free (SPF) conditions at a controlled temperature of 22 ± 2 °C, relative humidity of 65 ± 10%, and a 12 h light/dark cycle [1]. Rats were provided with ad libitum access to a standard laboratory chow diet (Guangdong Provincial Laboratory Animal Center, sterilized by irradiation) and autoclaved drinking water. After a 7-day acclimatization period, animals were randomly allocated to either the control group or the nano-plastics (NPs) exposure group, with eight mice per group (two cages per group, four rats per cage).

**Microplastics preparation and exposure**

Green-fluorescent polyethylene terephthalate microplastics (PET-NPs) with an average particle diameter of 200 nm (Huge Biotechnology, China) were used. Particle size distribution and morphology were verified by scanning electron microscopy (SEM) prior to use (Figure S16). PET-NPs suspensions were sonicated using a probe-type ultrasonic processor at 40 kHz and 120 W for 15 min in pulse mode (3 s on / 2 s off) to prevent overheating and aggregation. Particle size and dispersion status were checked immediately after sonication using dynamic light scattering (DLS). For exposure, PET-NPs were suspended in sterile distilled water at a final concentration of 15 μg/mL, corresponding to an estimated daily intake of ~150 μg/mouse/day (assuming an average water consumption of 10 mL/day and a body weight of ~27 g), i.e. approximately 5–6 mg/kg bw/day [2]. The suspension was freshly prepared every 24 h and ultrasonicated for 15 min before use to avoid particle aggregation. Rats in the NPs group received PET-NPs suspension as their sole drinking water source for 9 consecutive weeks, whereas control rats received sterile distilled water. Rats were marked with 0.5% neutral red solution for individual identification. Body weight was recorded weekly, and food and water intake were measured daily at the cage level. General health and behavior (locomotion, grooming, social interaction) were monitored daily by blinded animal technicians. All procedures were conducted in accordance with the National Institutes of Health Guide for the Care and Use of Laboratory Animals (8th edition) and approved by the Institutional Animal Care and Use Committee of the Nanjing Medical University (Approval No.: IACUC-2510049).

**Sample collection**

At the end of week 9, rats were fasted overnight, anesthetized with isoflurane, and blood was collected via cardiac puncture into EDTA-coated tubes [3]. Plasma was separated by centrifugation (3,000 × g, 10 min, 4 °C) and stored at −80 °C. After euthanasia by exsanguination, the liver, kidneys, colon, and epididymides were excised, blotted dry, weighed, and either fixed in 4% paraformaldehyde for histology or snap-frozen in liquid nitrogen for biochemical and molecular analyses [4]. Colon length was measured from the ileocecal junction to the anus. The anatomical process and sample collection after 9 weeks of exposure are shown in Figure S17.

Plasma biochemical parameters—including glucose, total cholesterol (TC), total bile acids (TBA), pyruvate, triglycerides (TG), and arginase—were measured using commercial assay kits (Table S5). Energy metabolism–related metabolites (PEP, GDP, ATP, GTP, NADP, R5P, ADP, GMP, for instance) and plasma bile acid profiles were quantified by targeted LC–MS/MS (Thermo Fisher Scientific, USA) with stable isotope–labeled internal standards [3]. Hepatic and renal TG and TC contents were determined using colorimetric enzymatic kits (Table S5). Enzyme activities in intestinal tissue homogenates—including superoxide dismutase (SOD), catalase (CAT), glutathione peroxidase (GSH-Px), myeloperoxidase (MPO), and levels of malondialdehyde (MDA), reduced glutathione (GSH), oxidized glutathione (GSSG), nitric oxide (NO), and reactive oxygen species (ROS)—were measured spectrophotometrically or fluorometrically using manufacturer-specified detection wavelengths (Table S6).

**Bile acid profiles in the liver and intestine**

Bile acids in liver tissues and intestinal contents are typically quantified using targeted liquid chromatography–tandem mass spectrometry (LC–MS/MS), which provides high sensitivity and allows accurate discrimination of individual primary, secondary, and conjugated bile acid species [3]. Briefly, liver tissue (20–50 mg) or intestinal content (20–40 mg) is homogenized in aqueous solvent containing isotope-labeled internal standards, followed by protein precipitation with methanol or acetonitrile. After centrifugation and filtration, the supernatant is analyzed by LC–MS/MS in negative electrospray ionization mode using multiple-reaction monitoring (MRM) for absolute quantification. Alternative methods such as enzymatic cycling assays or ELISA kits can measure total bile acid levels, but they lack the specificity required to resolve individual bile acid species; therefore, LC–MS/MS remains the gold-standard approach for mechanistic studies of hepatic–intestinal bile acid metabolism.

**Histology and immunohistochemistry**

Paraffin-embedded sections (4 μm) of liver, kidney, and colon were stained with Alcian blue-Periodic acid (AB&PAS) following standard protocols to assess histopathological changes [5]. Immunohistochemical analysis was performed exclusively to detect Caspase-3 protein expression in kidney tissues [6]. Briefly, sections were deparaffinized, rehydrated, subjected to heat-induced epitope retrieval in citrate buffer (pH 6.0), blocked with 5% bovine serum albumin (BSA) for 30 min at room temperature, and incubated overnight at 4 °C with a primary anti–Caspase-3 antibody (GK500505A, Gene Tech). After washing, sections were incubated with HRP-conjugated secondary antibodies IgG for 1 h, and signals were visualized with a DAB substrate kit. Hematoxylin was used for counterstaining. Images were captured using an Olympus BX53 microscope, and staining intensity was quantified using ImageJ software (NIH, USA).

**Gene expression analysis**

Total RNA was extracted from liver, kidney, epididymides, and intestinal tissues using TRIzol reagent (Invitrogen, USA) and reverse-transcribed with a PrimeScript RT kit (Takara, Japan) [7,8]. Quantitative PCR (qPCR) was performed using SYBR Green Master Mix (Applied Biosystems, USA) on a QuantStudio 6 Flex Real-Time PCR System (Applied Biosystems, USA). Target genes included those related to hepatic lipid metabolism (*Chrebp*, *Pkm*, *Gk*, *Fat*, *Fatp2*, *Ppara*, *Cpt1a*, *Pparg*, *Fas*, *Dgat1*, *Dgat2*, *Gpat*), inflammation and immunity (*KbP6*5, *Nlrp3*, *IL-1β*, *IL-6*), intestinal mucus secretion (*Muc1*, *Muc2*, *Muc3*, *Klf4*, *Meprin-β*, *Retnlb*), and intestinal barrier function (*ZO1*, *CLDN2*, *OCLN*, *ALPI*, *SLC15A1*). In addition, epididymal tissue expression of *AC1*, *ACC*, *Dgat1*, *Dgat2*, and *GPa* was quantified to assess lipid metabolism in reproductive tissues. *GAPDH* served as the internal control. Primer sequences are provided in Table S7.

**Gut microbiome analysis**

Intestinal content samples were collected from the distal ileum and cecum under aseptic conditions immediately after euthanasia and snap-frozen in liquid nitrogen. Microbial genomic DNA was extracted using the QIAamp Fast DNA Stool Mini Kit (Qiagen, Germany) following the manufacturer’s protocol, with an additional bead-beating step (0.1 mm zirconia/silica beads, BioSpec, USA) for 3 × 60 s at 6.0 m/s using a FastPrep-24 instrument (MP Biomedicals, USA) to ensure efficient lysis of gut microbiome [9]. DNA concentration and purity were assessed by NanoDrop 2000 spectrophotometer (Thermo Fisher Scientific, USA) and agarose gel electrophoresis. Metagenomic libraries were constructed using the NEBNext® Ultra™ II DNA Library Prep Kit (New England Biolabs, USA) according to the manufacturer’s instructions [10]. Paired-end sequencing (2 × 150 bp) was performed on an Illumina NovaSeq 6000 platform (PE150) by Biomarker Technologies (Beijing, China). Raw reads were processed by removing adapters and low-quality sequences using Trimmomatic (v0.39), followed by host sequence removal via alignment to the mouse genome (GRCm39) using Bowtie2 (v2.4.5). High-quality clean reads were assembled de novo using MEGAHIT (v1.2.9), and open reading frames (ORFs) were predicted with Prodigal (v2.6.3). Taxonomic annotation was performed against the NCBI non-redundant (NR) protein database using DIAMOND (v2.0.15), and functional annotation was conducted using the KEGG, eggNOG, and CAZy databases [4,11].

**Statistical analysis**

Data normality was formally assessed using the Shapiro–Wilk test. Continuous variables conforming to a normal distribution are presented as mean ± standard deviation (SD). For comparisons between two groups, unpaired two-tailed Student’s t-tests were used. For longitudinal data, Two-way Repeated Measures ANOVA was performed to assess the effects of treatment, time, and their interaction, followed by Bonferroni’s post-hoc test. To control for false positives, *p*-values for all multiple comparisons (including biochemical indices and correlation analyses) were adjusted using the Benjamini-Hochberg False Discovery Rate (FDR) method. An adjusted *p*-value (FDR) < 0.05 was considered statistically significant. All statistical analyses were performed using GraphPad Prism 9.0 (GraphPad Software, USA) and R software (version 4.3.1) [12].

Microbial community composition and diversity analyses were performed in R using the phyloseq and vegan packages. Alpha diversity indices (Shannon, Simpson, Chao1) and beta diversity metrics (Bray–Curtis dissimilarity, unweighted UniFrac distances) were calculated and visualized by principal coordinates analysis (PCoA). The statistical significance of β-diversity clustering was evaluated using Permutational Multivariate Analysis of Variance (PERMANOVA) with 999 permutations. Linear discriminant analysis effect size (LEfSe) was applied to identify differentially abundant taxa, with an LDA score > 3.0 and FDR < 0.05 considered significant. Phylogenetic trees were constructed with ggtree, and heatmaps were generated using pheatmap [13]. Correlation analyses between microbial taxa and host biochemical parameters were performed using Spearman’s correlation; only robust associations (|r| > 0.6) with statistical significance (*p* < 0.05) were visualized as network graphs using igraph (v2.2.1) and ggraph (v2.2.2) [14].

**References**

1. Xu, Xinming, Qingying Feng, Tao Zhang, Yunlong Gao, Qu Cheng, Wanqiu Zhang, Qinglong Wu, et al. 2024. “Infant Age Inversely Correlates with Gut Carriage of Resistance Genes, Reflecting Modifications in Microbial Carbohydrate Metabolism During Early Life.” *iMeta* 3: e169. <https://doi.org/https://doi.org/10.1002/imt2.169>

2. Garcia, Marcus M., Aaron S. Romero, Seth D. Merkley, Jewel L. Meyer-Hagen, Charles Forbes, Eliane El Hayek, David P. Sciezka, et al. 2024. “In Vivo Tissue Distribution of Polystyrene or Mixed Polymer Microspheres and Metabolomic Analysis after Oral Exposure in Mice.” 132: 047005. <https://doi.org/doi:10.1289/EHP13435>

3. Liang, Boxuan, Yanhong Deng, Yuji Huang, Yizhou Zhong, Zhiming Li, Jiaxin Du, Rongyi Ye, et al. 2024. “Fragile Guts Make Fragile Brains: Intestinal Epithelial Nrf2 Deficiency Exacerbates Neurotoxicity Induced by Polystyrene Nanoplastics.” *ACS Nano* 18: 24044−24059. <https://doi.org/10.1021/acsnano.4c03874>

4. Sun, Jiaqi, Miaomiao Teng, Wentao Zhu, Xiaoli Zhao, Lihui Zhao, Yunxia Li, Zixuan Zhang, Yunjie Liu, Sheng Bi, Fengchang Wu. 2024. “Microrna and Gut Microbiota Alter Intergenerational Effects of Paternal Exposure to Polyethylene Nanoplastics.” *ACS Nano* 18: 18085−18100. <https://doi.org/10.1021/acsnano.4c06298>

5. Liu, Yujing, Qiang Zhang, Lu Lu, Yufan Qian, Yuanmin Wu, Dan Hu, Yangxian Xu, Hanchen Xu, Guang Ji. 2025. “Huang-Qin Decoction Alleviates Deoxycholic Acid-Induced Colorectal Cancer in Mice by Regulating Gut Microbiota.” *Journal of Ethnopharmacology* 346: 119715. <https://doi.org/https://doi.org/10.1016/j.jep.2025.119715>

6. Lan, Shanshan, Bing Yang, Francis Migneault, Julie Turgeon, Maude Bourgault, Mélanie Dieudé, Héloïse Cardinal, Michael J. Hickey, Natacha Patey, Marie-Josée Hébert. 2021. “Caspase-3-Dependent Peritubular Capillary Dysfunction Is Pivotal for the Transition from Acute to Chronic Kidney Disease after Acute Ischemia-Reperfusion Injury.” *Renal Physiology* 321: F335−F351. <https://doi.org/10.1152/ajprenal.00690.2020>

7. Hsu, Wei-Hsuan, You-Zuo Chen, Yi-Ting Chiang, Yi-Tsen Chang, Yi-Wen Wang, Kung-Ting Hsu, Yi-Yun Hsu, Pei-Ting Wu, Bao-Hong Lee. 2025. “Polystyrene Nanoplastics Disrupt the Intestinal Microenvironment by Altering Bacteria-Host Interactions through Extracellular Vesicle-Delivered Micrornas.” *Nature Communications* 16: 5026. <https://doi.org/10.1038/s41467-025-59884-y>

8. He, Chengxun, Peipei Wang, Yuanyuan Zuo, Juan Guo, Qing Zhang, Shuyang Peng, Hong Zhang, Xueping Li, Chunjie Wu, Wei Peng. 2025. “Constituents-Composed Chinese Medicine of Dajianzhong Decoction Ameliorates Non-Alcoholic Fatty Liver Disease Via Ampk/Pparα/Cpt1 Signaling.” *Phytomedicine* 149: 157515. <https://doi.org/https://doi.org/10.1016/j.phymed.2025.157515>

9. Fernández-Pato, Asier, Trishla Sinha, Ranko Gacesa, Sergio Andreu-Sánchez, Milla F. Brandao Gois, Jody Gelderloos-Arends, Dianne B. H. Jansen, et al. 2024. “Choice of DNA Extraction Method Affects Stool Microbiome Recovery and Subsequent Phenotypic Association Analyses.” *Scientific Reports* 14: 3911. <https://doi.org/10.1038/s41598-024-54353-w>

10. Gand, Mathieu, Bram Bloemen, Kevin Vanneste, Nancy H. C. Roosens, Sigrid C. J. De Keersmaecker. 2023. “Comparison of 6 DNA Extraction Methods for Isolation of High Yield of High Molecular Weight DNA Suitable for Shotgun Metagenomics Nanopore Sequencing to Detect Bacteria.” *BMC Genomics* 24: 438. <https://doi.org/10.1186/s12864-023-09537-5>

11. Esther, Wortmann, Wylensek David, Basic Marijana, Hermeling Sven, Bleich André, Haller Dirk, Tolba René, Liebisch Gerhard, Janssen Klaus-Peter, Clavel Thomas. 2024. “Gut Microbiota Prevents Small Intestinal Tumor Formation Due to Bile Acids in Gnotobiotic Mice.” *Microbiome Research Reports* 3: 44. <https://doi.org/10.20517/mrr.2024.20>

12. Damian Riina, Matthew, Cassandra Stambaugh, Nathaniel Stambaugh, Kathryn E. Huber. 2023. Chapter 28 - Continuous Variable Analyses: T-Test, Mann–Whitney, Wilcoxin Rank. *Translational Radiation Oncology* Academic Press, 153−163. <https://doi.org/https://doi.org/10.1016/B978-0-323-88423-5.00070-4>

13. Xu, Shuangbin, Lin Li, Xiao Luo, Meijun Chen, Wenli Tang, Li Zhan, Zehan Dai, Tommy T. Lam, Yi Guan, Guangchuang Yu. 2022. “Ggtree: A Serialized Data Object for Visualization of a Phylogenetic Tree and Annotation Data.” *iMeta* 1: e56. <https://doi.org/https://doi.org/10.1002/imt2.56>

14. Xu, Zhimin, Yuxue Zhang, Zhihao Xu, Yuming Zhong, Lei Wang, Chunguang Liu, Ailing Chen, et al. 2023. “Dysregulation of Gut Health in Zebrafish by Differentially Charged Nanoplastic Exposure: An Integrated Analysis of Histopathology, Immunology, and Microbial Informatics.” *Environmental Science: Nano* 10: 933−947. <https://doi.org/10.1039/D2EN00542E>

**Supplementary figures**

**Figure S1 Effects of chronic NPs exposure on growth performance, organ indices, and systemic metabolic profiles in mice.** (A) Experimental design of the 9 — week NPs exposure, in which mice received 200 — nm NPs in drinking water followed by physiological and biochemical assessments. (B) Behavioral performance after chronic exposure. (C) Weekly food intake and water consumption during the exposure period. (D) Dynamic changes in body weight over 9 weeks. (E) Colon length and weight, and liver and kidney weights at the end of exposure. (F) Plasma biochemical indicators, including glucose (Glu), total cholesterol (TC), total bile acids (TBA), pyruvate (PYR), triglycerides (TG), and arginase (ARG). (G) Principal component analysis (PCA) and bubble heatmap of serum energy-related metabolites following NPs exposure. Data represent biological replicates and are shown as means (n = 3). *, ** indicate *p* < 0.05 and *p* < 0.001 versus control, respectively. Data are presented as mean ± SEM. Differences between the two groups were assessed by two-tailed Student's t-test.

**Figure S2 Co-occurrence network among TC and TG content, lipid metabolism-related genes, and inflammatory factors in liver and kidney after NPs exposure.** Correlation networks were constructed using the SparCC algorithm. Edges represent robust correlations with a correlation coefficient |r| > 0.6 and a statistical significance of FDR-adjusted *p* < 0.05.

**Figure S3 Chronic NPs exposure disrupts intestinal redox balance, barrier integrity, and inflammatory signaling.** (A and B) Immunofluorescence images of intestinal tissues after 9 - week NPs exposure, showing green fluorescence–labeled NPs and corresponding quantitative intensity analysis. (C) Representative H&E-stained intestinal sections demonstrating histopathological changes following chronic exposure. (D) Oxidative stress markers and antioxidant enzyme activities in intestinal tissues, including SOD, CAT, MDA, MPO, GSH, GSSG, GSH-PX, ROS, and NO. (E) Relative expression of mucus secretion – related genes (*Muc1*, *Muc2*, *Muc3*, *Klf4*, *Meprin-β*, and *Retnlb*). (F) Relative expression of barrier- and inflammation-associated genes (*ZO1*, *CLDN2*, *OCLN*, *ALPI,* *SLC15A1*, *NLRP3*, and *IL-6*). (G) Bubble heatmap showing correlations between intestinal functional genes and antioxidant enzyme activities. Data represent biological replicates and are shown as means (n = 3). * *p* < 0.05, ** *p* < 0.001 versus control. Data are presented as mean ± SEM. Differences between the two groups were assessed by two-tailed Student's t-test.

**Figure S4 Alpha diversity of intestinal bacterial communities in mice following chronic NPs exposure.** Alpha-diversity indices comparing control and NPs - exposed groups after 9 - week exposure. Different lowercase letters indicate significant differences at *p* < 0.05. Statistical significance was assessed using two-tailed Student’s t-test.

**Figure S5 Neutral community models illustrating the contribution of stochastic processes to community assembly after 9 - week NPs exposure.** (A and B) Assembly mechanisms of bacterial communities in the CK and NPs groups. (C and D) Assembly mechanisms of fungal communities in the CK and NPs groups. (E and F) Assembly mechanisms of viral communities in the CK and NPs groups.

**Figure S6 Heat-tree visualizations showing differential taxonomic abundance of bacterial, fungal, and viral communities between CK and NPs - exposed groups. (**A) Differential heat tree of bacterial communities. (B) Differential thermal trees of fungal communities. (C) Virus community differential hot tree. Statistical significance was assessed using two-tailed Student’s t-test.

**Figure S7** **Chronic NPs exposure alters intestinal bacterial community structure in mice.** PCoA showing global shifts in intestinal bacterial communities after 9 - week NPs exposure, Statistical significance was determined using PERMANOVA based on Bray - Curtis distances. Venn diagrams indicate shared and unique taxa between control and NPs groups. Relative abundances of the top eight bacterial families are displayed for both groups. Phylogenetic profiling of the top 60 bacterial species is shown, where the outer color squares represent min–max–normalized abundance (deep green = higher abundance), inner symbol size reflects log10 - transformed relative abundance, and central connecting lines (Use Spearman's method) denote interspecies correlation strength (green = positive correlations; black = negative correlations).

**Figure S8 Linear discriminant analysis effect size (LEfSe) identifying differentially enriched taxa after NPs exposure.** (A) Significantly enriched bacterial taxa. (B) Significantly enriched fungal taxa. (C) Significantly enriched viral taxa. LDA score > 4; FDR < 0.05.

**Figure S9** **Heatmap showing Spearman correlation coefficients between gut physicochemical indices and abundances of characteristic microbial taxa across bacterial, fungal, and viral communities.** OTU prefixes "V", "F" and "B" on the abscissa represent viruses, fungi and bacteria, respectively. **p* < 0.05, ***p* < 0.001 indicate statistically significant correlations. Statistical significance for group comparisons was assessed using Student’s t-test, and correlations were determined by Spearman’s rank correlation.

**Figure S10 Alpha diversity of intestinal fungal communities in mice following chronic NPs exposure.** Alpha-diversity indices comparing control and NPs-exposed groups after 9-week exposure. Different lowercase letters indicate significant differences at *p* < 0.05. Statistical significance was assessed using two-tailed Student’s t-test.

**Figure S11** **Chronic NPs exposure alters intestinal fungal community structure in mice.** PCoA showing global differences in intestinal fungal communities after 9 - week NPs exposure, Statistical significance was determined using PERMANOVA based on Bray - Curtis distances. Venn diagrams depict shared and unique fungal taxa between control and NPs groups. Relative abundances of the top eight fungal families are shown for both groups. Phylogenetic profiling of the top 50 fungal species is presented, where outer color squares represent min–max–normalized abundance (deep green = higher abundance), inner symbol size indicates log₁₀-transformed relative abundance, and central connecting lines (Use Spearman's method) reflect interspecies correlation strength (yellow = positive correlations; blue = negative correlations).

**Figure S12 Alpha diversity of intestinal viral communities in mice following chronic NPs exposure.** Alpha-diversity indices comparing control and NPs-exposed groups after 9-week exposure. Different lowercase letters indicate significant differences at *p* < 0.05. Statistical significance was assessed using two-tailed Student’s t-test.

**Figure S13 Chronic NPs exposure alters intestinal viral community structure in mice.** PCoA showing global differences in intestinal viral communities after 9 - week NPs exposure, Statistical significance was determined using PERMANOVA based on Bray - Curtis distances. Venn diagrams illustrate shared and unique viral taxa between control and NPs groups. Relative abundances of the top eight viral families are shown for both groups. Phylogenetic profiling of the top 50 viral species is presented, where outer color squares represent min–max–normalized abundance (deep green = higher abundance), inner symbol size indicates log₁₀-transformed relative abundance, and central connecting lines (Use Spearman's method) denote interspecies int

**Figure S14 GO functional enrichment of intestinal microbiota in mice following chronic NPs exposure.** Gene Ontology (GO) enrichment analysis of intestinal microbiota after 9-week NPs exposure. Top10 up-regulated and down-regulated GO functions were selected respectively (*p* < 0.05). Yellow indicates Biological Process, red indicates Cellular Component, and green indicates Molecular Function categories.

**Figure S15 Effects of chronic NPs exposure on intestinal carbohydrate-active enzymes (CAZymes) and antibiotic resistance genes (ARGs).** (A, B and H) Chord diagrams illustrating relative expression patterns of CAZyme families (GH, GT, PL, CE, AA, and CBM) and the top 10 ARGs in control and NPs-exposed groups. (C, D and I) Proportional distributions of CAZyme families and ARG categories between treatments. (E and J) Sankey plots showing significantly enriched carbon metabolism pathways and ARGs (*p* < 0.05), highlighting the top five microbial contributors; bar lengths represent log₁₀-transformed relative abundance. (F and K) Bubble plots of differentially expressed carbon metabolism–related genes and ARGs (absolute fold change > 1.5). (G) PCoA of ARG profiles, with different lowercase letters indicating significant differences among groups. Data represent biological replicates and are shown as means.

**Figure S16 SEM and fluorescence images characterizing the tested NPs.** The 200 nm particles were loaded with approximately 10% imported fluorescent dye molecules embedded within the internal matrix, ensuring high-intensity, stable, and concentrated fluorescence emission. The physicochemical properties of the particles include a density of 1.05 g/cm³ and a refractive index of 1.59 at 589 nm (25°C).

**Figure S17** **The dissection process and sample collection of mice after 9 weeks of exposure.** Mice were anesthetized, and physiological parameters (body length and body weight) were recorded prior to dissection, followed by the determination of organ parameters.
